# The Role of Angiotensin II Type 1 Receptor A1166C Polymorphism in Autosomal Dominant Polycystic Kidney Disease

**DOI:** 10.7759/cureus.41136

**Published:** 2023-06-29

**Authors:** Anand Sasidharan, Bhargavi MV, Rajkumar Mani, Sathyamurthy P

**Affiliations:** 1 General Medicine, Sri Ramachandra Institute of Higher Education and Research, Chennai, IND

**Keywords:** angiotensin ii type 1 receptor, angiotensin receptor type 1 - at1r, adpkd, a1166c gene polymorphism, at1r gene polymorphism, autosomal dominant polycystic kidney

## Abstract

Introduction

Autosomal dominant polycystic kidney disease (ADPKD) is the most common monogenic disorder that affects the kidney, which affects all ethnical groups worldwide, with varied clinical presentations and severity. The studies done in various parts of the world on the association between angiotensin II type 1 receptor (AT1R) A1166C gene polymorphism and ADPKD patients have revealed inconsistent results. This study was done to assess the role of AT1R A1166C gene polymorphism in ADPKD in the South Indian population, which is the first of its kind.

Methodology

This is a case-control study, conducted at a tertiary care center in South India. This study was concerned with the frequency of exposure (genotype) in ADPKD patients. Peripheral blood samples from 85 unrelated ADPKD patients and 94 controls without diabetes, hypertension, or any kidney-related disease were collected. The AT1R A1166C polymorphism was compared between (i) the cases and controls, (ii) early and late stages of chronic kidney disease (CKD) (ADPKD) subjects, and (iii) ADPKD subjects with and without hypertension.

Results

Among the ADPKD patients, 45 (52.9%) subjects showed early stage (CKD stages 1-3), and 40 (47%) subjects showed late stage CKD (CKD stages 4 and 5). The genotype distribution of the studied 85 ADPKD patients was almost similar. No significant association was found between the genotype distribution of AT1R A1166C polymorphism in AA vs. AC (OR = 1.11; 95% CI = 0.37-3.32; p < 0.844) and A vs. C (OR = 1.11; 95% CI = 0.38-3.32; p < 0.847) between cases and controls. The genotype distributions in genetic model AA vs*.* AC (OR = 3.07; 95% CI = 0.56-16.8; p < 0.177) and allelic model A vs. C (OR = 2.13; 95% CI = 0.40-11.3; p < 0.364) between the early and late CKD stages of ADPKD were also not significant. No significant association of gene polymorphism was found between the non-hypertensive and hypertensive groups of ADPKD.

Conclusion

The results of our study suggest there is no significant association between AT1R A1166C polymorphisms and ADPKD in the South Indian population. Further, the gene polymorphism is not related to the progression of ADPKD or the presence of hypertension in ADPKD cases in South India.

## Introduction

Autosomal dominant polycystic kidney disease (ADPKD) is a multi-system disorder and the most common monogenic disorder that affects the kidney. It affects all ethnical groups worldwide, with an estimated frequency of 1:500 to 1000 [1], where multiple cysts filled with fluid develop in both kidneys progressively. Microalbuminuria and hypertension increase and the glomerular filtration rate decreases progressively in the course of the disease, leading to end-stage renal disease (ESRD) in one-half of the patients after 50 years of age [2]. Extra-renal manifestations in ADPKD are aneurysms or cysts in the liver, seminal vesicle, pancreas, spleen or lung, cerebral aneurysms, cardiac valve lesions, and colonic diverticula.

The severity of the disease, age at onset of ESRD, and the extra-renal manifestations vary widely even between the affected individuals of the same family [3], indicating the need for identification of a novel gene polymorphism, which could lead to improved diagnostic accuracies and therapeutic options.

The role of renin-angiotensin in hypertension and the progression of chronic kidney disease (CKD) is well known. Hypertension is present in around 60% of ADPKD patients even before the glomerular filtration rate starts declining, denoting that it is a crucial risk factor in the progression of kidney failure [4]. The angiotensin II type 1 receptor (AT1R) gene, which is widely expressed in kidneys, smooth muscles of blood vessels, and multiple other human tissues, is linked with high blood pressure (BP) and the progression of renal disease [5,6].

The most well-studied polymorphism, AT1R A1166C (rs5186), has shown a faster decline in glomerular filtration rate in ADPKD in Argentinians [7] and an association of the C allele with faster progression to ESRD than the A allele in the Polish population [8]. But two different studies done in the East Asian population did not reveal any correlation between AT1R A1166C polymorphism and ADPKD [9,10]. There are no available studies in the South Indian population in relation to the role of AT1R gene polymorphisms in CKD progression among ADPKD individuals and the association between AT1R gene polymorphisms and hypertension in ADPKD patients. Hence, this study was intended to study the role of AT1R gene polymorphisms in ADPKD patients in the South Indian population, and its association with CKD progression and presence of hypertension.

## Materials and methods

This is a case-control study. Patients with ADPKD in various stages, visiting our center, a tertiary care teaching hospital located in Chennai, for treatment, were the case samples. All participants were more than 18 years of age, with established ADPKD diagnosis, with or without type 2 diabetes. Patients with other causes of CKD or type 1 diabetes mellitus were excluded. Subjects without diabetes, hypertension, or any kidney-related disease were included as controls.

The sample size was calculated using the Power and Sample Size Calculation program (version 2.1.31). Based on a power analysis, 85 unrelated ADPKD patients and 94 control subjects were chosen. Procedures for the protection of human subjects in this study were approved by the Institutional Ethical Review Committee of Sri Ramachandra University, Chennai (IEC-NI/09/MAR/08/09). Written informed consent was collected from the study participants before collecting the blood samples.

The study participants were examined and blood pressure was measured with a sphygmomanometer. The average of the three blood pressure values was taken. Blood samples were collected for hemoglobin, blood urea nitrogen, creatinine, sodium, potassium, bicarbonate, and calcium. The estimated glomerular filtration rate (eGFR) was calculated with the Modification of Diet in Renal Disease (MDRD) formula and CKD was staged on the basis of Kidney Disease Outcomes Quality Initiative (KDOQI) guidelines. The ADPKD patients were split into two groups: early (stages 1-3 of CKD) and late (stages 4 and 5 of CKD). Further classification of cases, based on the presence or absence of hypertension, was also made.

From the collected intravenous blood samples of study participants, DNA was extracted by a modification of the salting out method of Miller et al., precipitated in ethanol, and re-suspended in 500 µL of Tris-ethylenediaminetetraacetic acid (EDTA) (TE) buffer [11]. The visual quantification of DNA was done and the quantified DNA was used for further analysis. For the determination of the concentration of the DNA in the sample, NanoDrop ND-1000 Spectrophotometer (Thermo Scientific, Nanodrop Technologies, Wilmington, DE) was used. A genomic DNA quality check was done by 0.8% Medox horizontal agarose gel electrophoresis. The gene polymorphism was detected by polymerase chain reaction-restriction fragment length polymorphism. The allele and genotype frequencies were analyzed by gene counting and comparison made with 2 × 2 and 2 × 3 contingency tables, respectively (accessible at http://faculty.vassar.edu/lowry/VassarStats.html). Hardy-Weinberg exact test was applied to assess the consistency of the observed and expected genotype frequency distribution, based on the Hardy-Weinberg equilibrium hypothesis. At any stage, if the observed or expected number in each cell was below five, Fisher’s exact test was applied. The Hardy-Weinberg equilibrium test and the comparison of allele and genotype distribution were done by chi-square test with one degree of freedom and Monte Carlo simulation test applying the HWSIM program. A p-value less than 0.05 was considered significant for all statistical tests. Clinical characteristics were compared with Student’s unpaired t-test and the χ2 test.

## Results

The case group had 85 subjects (58.8% male and 41.1% female) with ADPKD and 94 (62.7% male and 37.2% female) healthy subjects not related to the case group were assigned to the control group. The mean age of the case group was 46.9 ± 10.4 years and the control group was 53.3 ± 12.4 years, and the difference was statistically significant (p < 0.001) (Abstract: Anand Sasidharan. Role of Angiotensin II Type 1 Receptor Polymorphism in Progression of Chronic Kidney Disease and Its Association With Hypertension Among Autosomal Dominant Polycystic Kidney Disease Patients. Mini-Orals of 58th European Renal Association - EDTA Virtual Congress; 5-8 June 2021).

In the case group, 40 (47%) subjects showed late CKD stage (eGFR < 30 mL/min/1.73 m2) with a mean age of 50.5 ± 10.0 years, and 45 (52.9%) subjects showed early stage (eGFR > 15 mL/min/1.73 m2) with a mean age of 44.1 ± 10.0 years and the difference was found to be statistically significant (p < 0.008) (Table 1). Based on the presence or absence of hypertension, the ADPKD case group was divided into two groups: non-hypertensives (n = 15) and hypertensives (n = 70). Among the studied characteristics, except sodium (p = 0.446) and chloride (p = 0.796), the differences in all other characteristics between the control and ADPKD groups were found to be statistically significant (p = 0.001) (Table 1) (Abstract: Anand Sasidharan, 5-8 June 2021).

**Table 1:** Clinical characteristics of study participants BUN: blood urea nitrogen; eGFR: estimated glomerular filtration rate.

Clinical characteristics	Controls (n = 94), Mean ± SD	ADPKD cases (n = 85), Mean ± SD	p-value
Age (years)	53.3 ± 12.4	46.9 ± 10.4	<0.001
BUN (mg/dl)	13.1 ± 6.7	18.0 ± 9.0	<0.001
Creatinine (mg/dl)	0.9 ± 0.2	3.0 ± 2.6	<0.001
Sodium (mmol/l)	136.7 ± 5.6	135.8 ± 9.0	0.446
Potassium (mmol/l)	3.8 ± 0.6	4.4 ± 2.3	0.024
Chloride (mmol/l)	101.3 ± 6.0	100.9 ± 11.8	0.796
Bicarbonate (mmol/l)	25.0 ± 3.5	22.8 ± 4.4	<0.001
Calcium (mg/dl)	10.3 ± 1.4	7.7 ± 1.2	<0.001
eGFR (ml/min/1.73 m^2^)	83.2 ± 21.4	43.6 ± 35.2	<0.001

AT1R A1166C polymorphism and ADPKD

**Figure 1:**
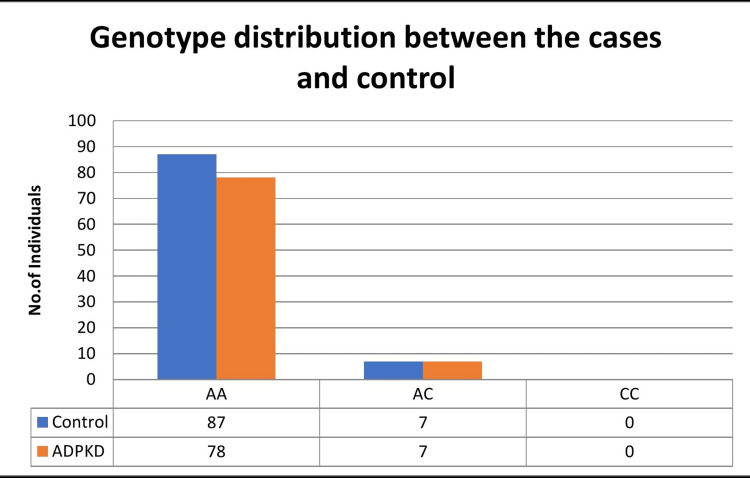
The genotype distribution between the ADPKD cases and controls ADPKD: autosomal dominant polycystic kidney disease.

The AT1R A1166C gene polymorphism was mapped for 85 ADPKD case subjects and 94 control subjects. The genotype dispersal of the 85 ADPKD subjects was AA = 78 (91.7%), AC = 7 (8.24%), and CC = 0 (0%) and that of 94 control subjects was AA = 87 (92.5%), AC = 7 (7.45%), and CC = 0 (0%) (Figure 1) (Abstract: Anand Sasidharan, 5-8 June 2021). The expected and observed genotype frequencies of both controls and cases of AT1R A1166C polymorphism were calculated and presented in Table 2. The Hardy-Weinberg equilibrium of control subjects (X2 = 0.14 and p = 0.707) and ADPKD cases (X2 = 0.156 and p > 0.692) did not deviate.

**Table 2:** Observed and expected genotypes of ADPKD cases and controls ADPKD: autosomal dominant polycystic kidney disease.

Control					Case				
Genotype	Observed	Expected	X^2^ value	p-value	Genotype	Observed	Expected	X^2^ value	p-value
AA	87	87.1	0.14	0.707	AA	78	78.1	0.156	0.692
AC	7	6.7			AC	7	6.7		
CC	0	0.1			CC	0	0.1		

The association between ADPKD patients and AT1R (A1166C) genotypes expressed as odds ratio, 95% confidence interval, and association chi-square test were calculated and documented in Table 3. The genotype distributions of AT1R A1166C polymorphisms in genetic model AA vs. AC (OR = 1.11; 95% CI = 0.37-3.32; p < 0.844) and allelic model A vs. C (OR = 1.11; 95% CI = 0.38-3.32; p < 0.847) between the ADPKD cases and controls were not found to be significant (Table 3) (Abstract: Anand Sasidharan, 5-8 June 2021).

**Table 3:** Association between ADPKD patients and AT1R (A1166C) genotypes ADPKD: autosomal dominant polycystic kidney disease; HWE: Hardy-Weinberg equilibrium; MAF: minor allele frequency. * Chi-square p-value.

Genotypes	Control (n = 94) (%)	ADPKD (n = 85) (%)	OR (95% CI), p-value*	
AA	87 (92.5)	78 (91.7)	Reference 1.11 (0.37-3.32) 0.844	
AC	7 (7.45)	7 (8.24)		
CC	0 (0)	0 (0)	-	-
A	181 (96.3)	163 (95.9)	Reference 1.11 (0.38-3.32) 0.847	
C	7 (3.7)	7 (4.1)		
MAF	3.72	4.12	-	-
HWE-p	0.707	0.692	-	-

AT1R A1166C polymorphisms and CKD stages

**Figure 2:**
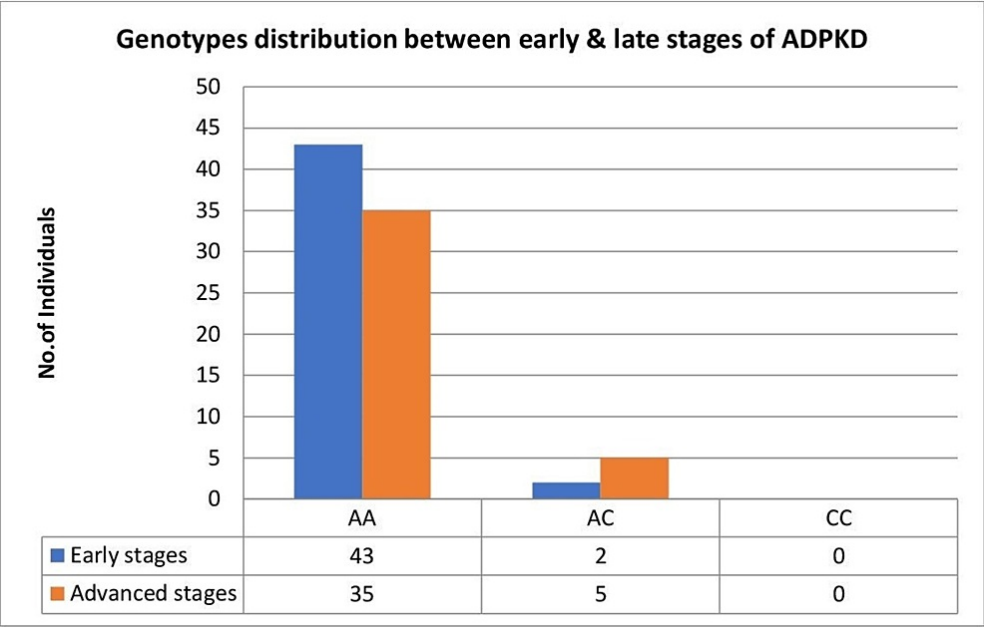
The AT1R A1166C genotype distribution between early and late stages of ADPKD patients ADPKD: autosomal dominant polycystic kidney disease.

Of the 85 case participants, 40 (47%) were in the late CKD stage group with a mean age of 50.5 ± 10.0 years, and 45 (52.9%) subjects showed early stage with 44.1 ± 10.0 years of mean age, which was statistically significant (p < 0.008). Of the 40 late-stage CKD patients, the genotype distribution was AA = 35 (87.5%), AC = 5 (12.5%), and CC = 0 (0%), and among the 45 early stages of CKD patients, the distribution was AA = 43 (95.5%), AC = 2 (4.4%), and CC = 0 (0%) (Figure 2) (Abstract: Anand Sasidharan, 5-8 June 2021).

The major homozygote as reference genotype, the odds ratio, 95% confidence interval, and association chi-square test were calculated for early and late CKD stages of ADPKD and documented in Table 4. The genotype distributions of AT1R A1166C polymorphisms in genetic model AA vs. AC (OR = 3.07; 95% CI = 0.56-16.8; p < 0.177) and allelic model A vs. C (OR = 2.13; 95% CI = 0.40-11.3; p < 0.364) were not found to be significantly associated between the early and late CKD stages of ADPKD (Abstract: Anand Sasidharan, 5-8 June 2021). The other characteristics, including age and gender, were also not significantly associated. However, the family history of diabetes mellitus was found to have a significant association (p = 0.001) between the early and late CKD stages of ADPKD (Table 4).

**Table 4:** AT1R A1166C gene polymorphism association between early and advanced stages of ADPKD patients ADPKD: autosomal dominant polycystic kidney disease; FH-DM: family history of diabetes mellitus.

Genotypes		Early stages (n = 45) (%)	Advanced stages (n = 40) (%)	OR (95% CI)	p-value
AA		43 (95.5)	35 (87.5)	3.07 (0.56-16.8)	0.177
AC		2 (4.4)	5 (12.5)		
CC		0 (0)	0 (0)	-	-
A		88 (97.8)	75 (93.8)	2.13 (0.40-11.3)	0.364
C		2 (2.2)	5 (6.3)		
Age in years	≤40	13 (28.9)	6 (15.0)		
	(40-60)	30 (66.7)	28 (70.0)	2.02 (0.67-6.05)	0.203
	>60	2 (4.4)	6 (15.0)	6.5 (1.00-42.1)	0.038
Gender	F	16 (35.6)	19 (47.5)	0.60 (0.25-1.45)	0.264
	M	29 (64.4)	21 (52.5)		
FH-DM	No	36 (80.0)	16 (40.0)	6.0 (2.28-15.76)	0.001
	Yes	9 (20.0)	24 (60.0)		

Association between AT1R A1166C polymorphism and hypertension in ADPKD patients

**Figure 3:**
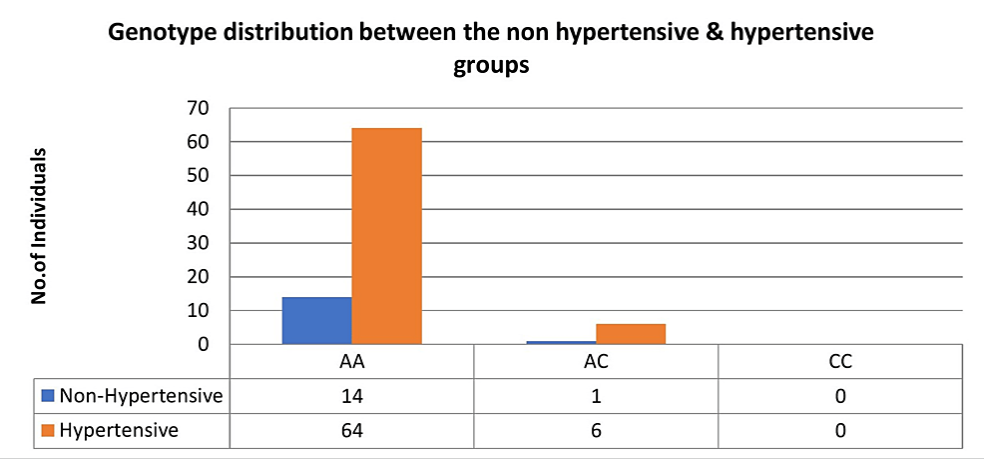
The genotype distribution between the non-hypertensive and hypertensive groups of ADPKD cases ADPKD: autosomal dominant polycystic kidney disease.

Further, based on the presence or absence of hypertension, the ADPKD cases were divided into two groups: non-hypertensives (n = 15), with a mean age of 48 ± 14.0 years, and hypertensives (n = 70), with a mean age of 46.6 ± 9.57 years. However, there was no statistically significant difference between the non-hypertensive and hypertensive groups (p = 0.734) of ADPKD patients. Of the 15 patients in the non-hypertensive group, the genotype distribution was AA = 14 (93.3%), AC = 1 (6.6%), and CC = 0 (0%), and among the 70 patients in the hypertensive group, the genotypes were AA = 64 (91.4%), AC = 6 (8.57%), and CC = 0 (0%) (Figure 3).

The major homozygote as reference genotype, the odds ratio, 95% confidence interval, and association chi-square test were calculated for hypertensive and non-hypertensive groups and documented in Table 5. The genotype distributions of AT1R A1166C polymorphism in genetic model AA vs. AC (OR = 1.31; 95% CI = 0.14-11.78; p < 0.867) and allelic model A vs. C (OR = 1.29; 95% CI = 0.15-11.2; p < 0.811) were not found to be significantly associated between the non-hypertensive and hypertensive groups of ADPKD. The other characteristics, including gender (p = 0.291) and family history of diabetes mellitus (p = 0.917), also did not show a significant association between the non-hypertensive and hypertensive groups of ADPKD (Table 5).

**Table 5:** The association of AT1R A1166C gene polymorphism between hypertensive and non-hypertensive individuals of ADPKD ADPKD: autosomal dominant polycystic kidney disease; FH-DM: family history of diabetes mellitus.

Genotypes		Non-hypertensive (n = 15) (%)	Hypertensive (n = 70) (%)	OR (95% CI)	p-value
AA		14 (93.3)	64 (91.4)	1.31 (0.14-11.78)	0.867
AC		1 (6.67)	6 (8.57)		
CC		0 (0)	0 (0)	-	-
A		29 (96.7)	134 (95.7)	1.29 (0.15-11.2)	0.811
C		1 (3.3)	6 (4.3)		
Age in years	≤40	4 (26.7)	15 (21.4)	Reference	
	40-60	8 (53.3)	50 (71.4)	1.66 (0.44-6.31)	0.448
	>60	3 (20.0)	5 (7.1)	0.44 (0.07-2.7)	0.373
Gender	Female	8 (53.3)	27 (38.6)	1.82 (0.59-5.59)	0.291
	Male	7 (46.7)	43 (61.4)		
FH-DM	No	9 (60.0)	43 (61.4)	0.94 (0.30-2.94)	0.917
	Yes	6 (40.0)	27 (38.6)		

## Discussion

Analysis of 85 ADPKD and 94 control subjects for AT1R A1166C polymorphism revealed that no significant association existed between the ADPKD patients and controls. Similarly, between the CKD stages (early and late) and hypertensive/non-hypertensive groups also, there was no statistically significant association. Our study revealed that the AT1R A1166C gene polymorphism does not have any impact on ADPKD, CKD progression, or hypertension in ADPKD patients.

Angiotensin II is the major biologically active product of the renin-angiotensin-aldosterone system (RAAS), formed from its original precursor angiotensinogen, by two successive enzymatic cleavages. Angiotensin II acts as a potent vasoconstrictor and exerts its effects through two structurally different receptor subtypes: angiotensin II type 1 receptor and type 2 receptor [12]. In humans, the AT1R is widely expressed in multiple tissues, including kidney and vascular smooth muscle cells, and is associated with increased blood pressure and progression of kidney disease [6]. The gene coding for AT1R is localized to chromosome 3q21q25, spans 45.123 kb, and comprises five exons. The first four exons represent the 5′-UTR, whereas exon 5 harbors the coding region. Several polymorphic sequence variants have been found on the AT1R gene. The most well-studied polymorphism is A1166C (rs5186), a transversion at position 1166, which is located in the 3′-UTR of the AT1R gene [13]. Reporter silencing assays have demonstrated that the 1166C allele interrupts the base-pairing complementarities of miR-155 and reduces the ability of miR-155 to interact with the cis-regulatory site. This indicates that the miR-155 downregulates the expression of the 1166A allele but not the 1166C allele. Thus, the likelihood of failure in the downregulation of AT1R expression as the biological base is most plausible.

The AT1R 1166-C allele has demonstrated an increased risk for coronary artery disease, ischemic stroke, heart failure, ESRD, and hypertension. It has been associated with glomerular filtration rate decline in ESRD caused by ADPKD in Argentinians [7]. Polish individuals with ESRD due to various causes have shown a higher frequency of combined AC and CC genotypes of A1166C polymorphisms. Furthermore, patients with the C allele have progressed to ESRD very quickly than the A allele [8]. In contrast to this, no association between the age of onset of ESRD and AT1R A1166C polymorphism has been observed in polycystic kidney disease 1 gene families in the United Kingdom and Australia [14]. Furthermore, two independent studies in East Asian populations have also failed to show an association between AT1R A1166C polymorphism and ADPKD [9]. Similarly, our study also observed that there was no association between the early and late stages of CKD in ADPKD patients of the South Indian population.

Evidence suggestive of the linkage of hypertension to the genetic area containing the AT1R gene has been confirmed with a logarithm of the odds score of 2.9 in a study from Finland by Kainulainen et al. [15]. Although no evidence of linkage has been found between A1166C and hypertension in the French population in a study by Bonnardeaux et al., a significant increase in C allelic frequency has been observed in hypertensives than the normotensive individuals [13]. A meta-analysis conducted on the Chinese population found that the AC/CC genotype of the AT1R gene was associated with a statistically increased essential hypertension risk with the pooled OR of 1.48 (95% CI: 1.20-1.83) [16].

In the Ohasama study, ambulatory blood pressure (ABP) monitoring in a general Japanese population of 802 Japanese subjects, aged 40 and above, found that the A/C1166 gene polymorphism of the AT1R gene was not associated with any clinical parameters associated with hypertension or atherosclerosis [17]. A prospective cohort study included 18,436 Caucasian women who were free of hypertension and did not receive antihypertensive drugs at baseline and found that AT1R A1166C polymorphisms were not consistently associated with incident hypertension or blood pressure progression [18]. A case-control study conducted in North India showed a significant association of A1166C polymorphism in the AT1R gene with essential hypertension and an increased expression of the AT1R gene C allele in patients with A1166C polymorphism [19]. A study performed in the South Indian Tamil population suggested that AT1R A1166C gene polymorphism is not associated with essential hypertension [20].

Studies thus far have not shown consistent evidence for the association between AT1R gene polymorphisms and hypertension among different races. In accordance with previous studies, our study also observed that there was no association between the AT1R A1166C polymorphisms and non-hypertensive/hypertensive groups in ADPKD patients.

However, this study has few limitations. First, we have not measured serum levels of AT1R to correlate it with gene polymorphisms. Second, the sample size was too small to conclude the results. Further studies are warranted with a large sample size covering multi-center/population with gold standard (sequencing) protocols to confirm our results. In lieu of applying next-generation sequencing technologies to study complex diseases, it is also possible to apply the same to unravel the complexity of CKD progression in ADPKD and hypertension-induced ADPKD.

## Conclusions

There was no significant association between AT1R A1166C gene polymorphism in ADPKD cases and control subjects in the South Indian population. There was no significant association found between the early and late CKD stages or between the non-hypertensive and hypertensive groups of ADPKD cases. However, the family history of type 2 diabetes mellitus and age of more than 60 years were found to be significantly associated with the CKD stages.
